# Meta-Analysis of the Association between Lumican Gene Polymorphisms and Susceptibility to High Myopia

**DOI:** 10.1371/journal.pone.0098748

**Published:** 2014-06-23

**Authors:** Miao He, Wei Wang, Dina Ragoonundun, Wenyong Huang

**Affiliations:** Zhongshan Ophthalmic Center, State Key Laboratory of Ophthalmology, Sun Yat-Sen University, Guangzhou, People's Republic of China; Casey Eye Institute, United States of America

## Abstract

**Backgrounds:**

Many studies have evaluated the association between lumican (LUM) gene polymorphisms and high myopia. However, the results remain controversial. This meta-analysis aims to comprehensively evaluate the relationship between two common LUM polymorphisms (rs3759223 and rs3759222) and the risk of high myopia.

**Methods:**

A comprehensive literature search for studies published up until September of 2013 was performed. Data were extracted independently by two investigators, and the weighted Odds Ratios (ORs) and 95% Confidence Intervals (CIs) for the associations were obtained by using a random-effects model.

**Results:**

Eight studies (1425cases and 1271 controls) were identified for the analysis of the association between rs3759223 polymorphism and high myopia. The results indicated that rs3759223 polymorphism was associated with high myopia under a recessive model (OR = 1.71, 95%CI 1.04–2.81). Further subgroup analysis indicated that this polymorphism was associated with high myopia among Chinese people in the additive model (OR = 1.17, 95%CI 1.06–1.29) and a recessive model (OR = 1.75, 95%CI 1.00–3.06) with control group coming from hospital based population. Four studies (1024 cases and 1163 controls) were identified for the analysis of the association between rs3759222 polymorphism and high myopia. The results indicated that rs3759222 polymorphism was not associated with high myopia in all genetic models, even the subgroup analysis couldn't provide relative proof to assure the outcome.

**Conclusion:**

This meta-analysis suggests that LUM polymorphisms are associated with the risk of high myopia. However, well-designed studies with larger sample sizes and more ethnic groups are required to further validate this association.

## Introduction

Myopia is a leading visual problem, with the prevalence of 82% in Asian countries and approximately 16% to 27% in western countries [1]–[3]. The public health impact, along with the associated costs of optical correction, is substantial. Myopic eyes with long axial lengths (≥26 mm) or high degrees of myopic refractive error (≤−6 D) are classified as high myopia [4]. The prevalence of high myopia is about 0.95% in China. High myopia differs from normal myopia in the increased possibility of various terrible accompanying complications, including chorioretinal degeneration, retinal detachment, and glaucoma [5]. High myopia is now considered the fourth most common cause of irreversible blindness [1], [6]. Therefore, it is important to elucidate the pathological mechanisms underlying high myopia.

Genetic associations with high myopia have been investigated for several decades [7]. Currently, several high myopia susceptibility genes have been identified, such as myocilin (MYOC), Hepatocyte Growth Factor (HGF), paired box gene 6 (PAX6), collagen type II alpha 1 (COL2A1), collagen type I alpha 1 (COL1A1), transforming growth factor beta 1 (TGFB1), Transforming Growth-Induced Factor (TGIF), and lumican (LUM) [7]. However, no definite pathogenetic gene has yet been found.

The LUM gene is located at 12q21 within a segment of the MYP3 gene (12q21–23) [8]. It is a member of the Small Leucine-Rich Proteoglycan (SLRP) gene family [9]. The SLRPs regulate collagen fibril formation and organization. Thus, they potentially influence the biomechanical properties of the sclera. Animal studies have shown that Lum^−/−^Fmod^−/−^ double-deficient mice demonstrate the physical signs of high myopia, including axial extension, scleral thinning, and retinal detachment, which suggest the LUM gene as a candidate gene for high myopia [8].

To date, many case-control studies have been carried out to investigate the role of LUM gene polymorphism in the development of high myopia [10]–[18]. However, these studies have yielded inconsistent results, especially concerning the SNP loci rs3759223 and rs3759222, which are the focus of significant current research interest. Hence, we performed a meta-analysis of all eligible studies to derive a more precise estimation of the association, helping us to better understand its possible influence on high myopia.

## Materials and Methods

This meta-analysis was performed according to a predetermined protocol described in the following paragraph. PRISM guidelines were followed at all stages of the process (Checklist S1) [19]. All stages of study selection, data extraction, and quality assessment were performed independently by two reviewers (W.W. and M.H). Any disagreement was resolved via discussion and consensus.

### 1. Literature search

Publications were identified through a systematic search of PubMed, Web of Science, EMBASE, and the Chinese Biomedicine Database (up to September of 2013). The keywords used were as follows: (polymorphism OR genotype OR variant OR allele OR variation OR SNP) AND (Lumican OR LUM) AND myopia. There were no limits placed on the year or language of publication. References identified from bibliographies of pertinent articles were also retrieved.

### 2. Study selection

The inclusion criteria were as follows: (1) studies were on the relationship between LUM gene polymorphism and high myopia; (2) case-control studies used either a Hospital-Based (HB) or a Population-Based (PB) design; (3) studies had full-text articles; (4) studies contained sufficient data for estimating an Odds Ratio (OR) within a 95% confidence interval (CI); and (5) studies did not republish data. Studies were excluded if they were family studies or published abstracts from meetings. If two or more studies shared the same cases or control subjects, the one with most informative was included. If more than one geographical or ethnic population were included in one article, each population was considered separately.

### 3. Data extraction and quality assessment

The following data were extracted from all qualified studies: first author's last name, publication year, population ethnicity, source of controls, age, definition of high myopia, study design, methods of genotyping, total numbers of cases and controls, and frequency of LUM gene polymorphism in both cases and controls. The qualities of the included studies were assessed independently by the same two investigators using the Newcastle-Ottawa Scale (NOS) [20]. Studies with NOS scores ≥7 were considered to be of high quality. Disagreements were settled as described above.

### 4. Statistical analysis

The Stata 11.0 software program examines the ORs and 95% CIs for four models: the allelic model (T allele vs. C allele), the additive model (T/T vs. C/C, T/T vs. T/C), the dominant model (T/T+T/C vs. C/C), and the recessive model (T/T vs. T/C+C/C) were used to assess the strength of association between rs3759223 polymorphism and high myopia. In the same way the allelic model (A allele vs. C allele), the additive model (A/A vs. C/C, A/A vs. A/C), the dominant model (A/A+A/C vs. C/C), and the recessive model (A/A vs. A/C+C/C) were used to assess the strength of association between rs3759222 polymorphism and high myopia. The Hardy-Weinberg equilibrium in controls was assessed using the chi-squared test. The data from individual studies were pooled by using the random-effect model, which considers within-study and between-study variation. Heterogeneity was assessed by using the Cochran Q and I^2^ statistics. For the Q statistic, a P value<0.10 was considered statistically significant for heterogeneity; for the I^2^ statistic, values of 25%, 50%, and 75% represent mild, moderate, and severe heterogeneity, respectively [21]. Because the potential causes of heterogeneity among studies were geographic region and design, a subgroup analysis were conducted on the basis of the various regions and designs. To evaluate the robustness of the results, each study in the meta-analysis was excluded in turn to expose the influence of the individual studies on the pooled estimates. Visual funnel plot inspection and statistical testing (Begg's and Egger's tests) were performed to evaluate the presence of publication bias [22], [23]. A P value<0.05 was considered significant, except where otherwise specified.

## Results

### 1. Literature search

**Figure 1:**
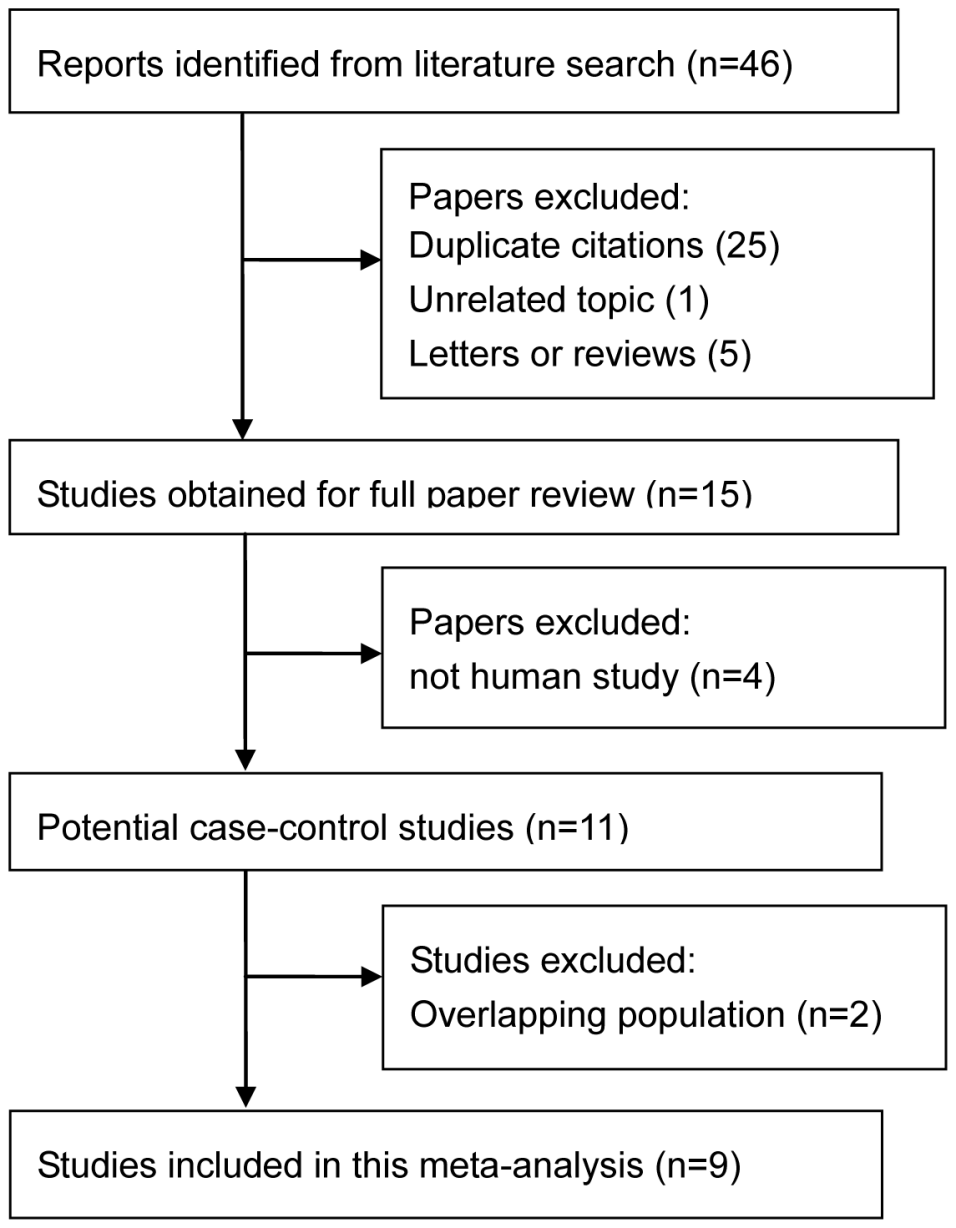
literature search flow.

The detailed steps of the study selection process are shown in Figure 1. Briefly, we initially identified 46 potentially eligible studies. Fifteen were considered as potentially relevant studies. Of these, four studies were excluded because they did not meet the inclusion criteria or were duplicate publications. This left us with nine case-control studies that met all inclusion criteria [10]–[18].

### 2. Characteristics of studies included

Table 1 shows the studies identified and their main characteristics. The publication year of the included studies ranged from 2006 to 2012. High myopia is defined as a refractive error ≤−6.00 D, ≤−6.50 D, ≤−8.00 D, ≤−9.25 D, and ≤−10.00 D in various studies. Controls were defined as subjects having no or mild myopia. Among the selected studies, one study was conducted in Korea, one in Hong Kong, three in Mainland China, and four in Taiwan. There were five PB studies and four HB studies. The genetic distributions of the control groups in all studies were consistent with HWE, except for one study [14]. The NOS results showed that the average score was 7.44(range 7 to 9), indicating that the methodological quality was generally good.

**Table 1:** Characteristics of case-control studies included in the meta-analysis.

First author (year)	Location	Source of control	Genotyping method	Definition of high myopia	Subjects (n)		Mean age(year)		HWE	NOS score
					Case	Control	Case	Control		
Park (2013)	Korea	PB	PCR-RFLP	≤−9.25 D	128	235	32.5	40.5	Yes	7
Dai (2012)	Mainland of China	PB	PCR-SSP	≤−10.00 D	292	299	NA	NA	Yes	8
Yip (2011)	Hong Kong	HB	PCR	≤−8.00 D	656	654	NA	NA	Yes	7
Lin(a) (2009)	Taiwan	PB	PCR	≤−6.50 D	182	79	18	18	Yes	7
Lin(b) (2009)	Taiwan	PB	PCR-RFLP, DS	≤−6.00 D	201	86	11	5	No	8
Chen (2009)	Taiwan	HB	Sequencing	≤−10.00 D	120	137	NA	NA	Yes	8
Zhang (2009)	Mainland of China	PB	PCR-DS	≤−6.00 D	94	90	37	36	Yes	8
Wang (a) (2009)	Mainland of China	HB	PCR-RFLP	≤−6.00 D	288	208	21.76	27.32	Yes	7
Wang (b) (2006)	Taiwan	HB	PCR-DS	≤−10.00 D	120	137	34.4	41.9	Yes	7

PB, population based; HB, hospital based; PCR, polymerase chain reaction; RFLP, restriction fragment length polymorphism; DS, direct sequencing; SSP, sequence-specific primer; D, diopter; NA, data not available; HWE, Hardy-Weinberg equilibrium in controls; NOS: Newcastle-Ottawa Scale.

### 3. Meta-analysis results

Eight studies (1425cases and 1271 controls) have evaluated the association between rs3759223 polymorphism and high myopia. There was a significant association between SNP rs3759223 and increased high myopia risk in the recessive model (OR = 1.71, 95%CI 1.04–2.81), but no significant association was present in the allelic model (OR = 1.32, 95%CI 0.89–1.96), the additive model (T/T vs. C/C OR = 1.74, 95%CI 0.71–4.26; T/C vs. C/C OR = 0.98, 95%CI 0.51–1.88) or the dominant model (OR = 1.21, 95%CI 0.59–2.52) (Table 2). There was substantial heterogeneity within these analyses. Further subgroup analysis indicated that this polymorphism was associated with high myopia in the population of mainland of China (additive model: OR = 1.17, 95%CI1.06–1.29) and HB subgroups (recessive model: T/T vs. C/C+T/C OR = 1.75, 95%CI 1.00–3.06) (Table 3).

**Table 2:** Results of the Meta-analysis for rs3759223 and rs3759222 polymorphism and high myopia.

Genotype contrast	Case	Control	OR	95%CI		Overall Effect		Heterogeneity	
			estimate	L	U	Z	P	I2(%)	P
rs3759223									
T vs C	2706	2144	1.32	0.89	1.96	1.39	0.166	89.00	0.000
TT vs CC	793	553	1.74	0.71	4.26	1.20	0.229	85.30	0.000
TC vs CC	674	569	0.98	0.51	1.88	0.06	0.955	80.80	0.000
(TT+TC) vs CC	1233	936	1.21	0.59	2.52	0.52	0.604	86.10	0.000
TT vs (CC+TC)	1233	936	1.71	1.04	2.81	2.13	0.003	81.90	0.000
rs3759222									
A vs C	1271	1535	1.13	0.96	1.32	0.85	0.133	0.00	0.855
AA vs CC	137	214	1.32	0.76	2.27	0.99	0.324	0.00	0.727
AC vs CC	404	531	1.13	0.80	1.59	0.68	0.495	0.00	0.337
(AA+AC) vs CC	248	372	1.17	0.84	1.62	0.93	0.353	0.00	0.368
AA vs (CC+AC)	248	372	1.27	0.76	2.11	0.90	0.371	0.00	0.953

**Table 3:** Results of the subgroup analysis for rs3759223 polymorphism.

Study group	T vs. C		T/T vs. C/C		T/C vs. C/C		T/T vs. T/C+C/C		T/T+T/C vs.C/C	
	OR(95%CI)	P	OR(95%CI)	P	OR(95%CI)	P	OR(95%CI)	P	OR(95%CI)	P
**rs3759223**										
**Region**										
Mainland of China	2.14(0.78–5.85)	0.138	3.81(0.61–23.93)	0.154	1.17(1.06–1.29)	0.002	2.31(0.84–6.40)	0.177	2.78(0.63–12.273)	0.313
Taiwan	0.99(0.82–1.20)	0.925	1.33(0.76–2.34)	0.321	0.78(0.68–0.80)	<0.001	1.48(0.73–3.00)	0.278	0.68(0.42–1.08)	0.001
Korea	1.03(0.70–1.51)	0.880	0.78(0.30–2.01)	0.604	0.93(0.79–1.11)	0.423	1.12(0.71–1.76)	0.523	0.74(0.29–1.88)	0.415
**Source**										
PB	1.48(0.74–2.95)	0.273	1.47(0.74–2.95)	0.438	0.99(0.91–1.09)	0.871	1.84(0.91–3.71)	0.090	1.27(0.41–3.92)	0.679
HB	1.15(0.90–1.46)	0.258	1.50(0.90–1.46)	0.258	0.92(0.80–1.05)	0.209	1.75(1.00–3.06)	0.048	1.09(0.38–3.13)	0.867

PB, population based; HB, hospital based; P, P value.

Four studies (1024 cases and 1163 controls) reported an association between rs3759222 polymorphism and high myopia. However, the pooled results indicated that there was no significant association between rs3759222 polymorphism and high myopia (Table 2). Further subgroup analysis also couldn't provide sufficient evidence to the support the positive relationship between rs3759222 polymorphism and high myopia. There was no evidence of heterogeneity for these analyses (Table 4).

**Table 4:** Results of the subgroup analysis for rs3759222 polymorphism.

Study group	A vs. C		A/A vs. C/C		A/C vs. C/C		A/C vs. A/C+C/C		A/A+A/C vs. C/C	
	OR(95%CI)	P	OR(95%CI)	P	OR(95%CI)	P	OR(95%CI)	P	OR(95%CI)	P
**rs3759222**										
**Region**										
Taiwan	1.04(0.77–1.39)	2.580	1.19(0.55–2.58)	0.652	0.93(0.55–1.57)	0.775	1.25(0.61–2.54)	0.546	1.19(0.55–2.58)	0.652
Hong Kong	1.13(0.90–1.41)	0.294								
Korea	1.26(0.90–1.76)	0.172	1.45(0.67–3.13)	1.450	1.30(0.83–2.05)	0.253	1.29(0.61–2.70)	0.507	1.45(0.67–3.13)	0.345
**Source**										
PB	1.26(0.90–1.76)	0.172	1.45(0.67–3.13)	0.345	1.16(0.91–1.48)	0.245	1.29(0.61–2.70)	0.507	1.33(0.86–2.05)	0.197
HB	1.09(0.91–1.31)	0.332	1.19(0.55–2.58)	0.652	0.97(0.76–1.23)	0.776	1.25(0.61–2.54)	0.546	1.26(0.92–1.71)	0.149

PB, population based; HB, hospital based; P, P value.

### 4. Sensitivity analysis

Sensitivity analyses were conducted to explore the source of this heterogeneity and to examine the influence of various exclusion criteria on the combined estimates. After excluding the study with the smallest sample size [16], the results remained the same, but no evidence of heterogeneity was observed in those genetic models (T vs. C: I^2^ = 0.00%, P = 0.481; T/T vs. C/C: I^2^ = 17.6%, P = 0.300; T/T+T/C vs. C/C: I^2^ = 45.70%, P = 0.100; T/T vs. T/C+C/C: I^2^ = 0.00%, P = 0.458) except T/C vs. C/C: I^2^ = 54.0%, P = 0.054. After the deletion of any other single study, the random-effect estimates were also substantially unchanged, suggesting the high stability of the meta-analysis results. The data are not shown but are available upon request.

### 5. Publication bias

Publication bias was qualitatively assessed via Begg's funnel plot and quantitatively assessed via Egger's test. Neither Begg's funnel plot nor Egger's test detected obvious evidence of publication bias in relation to any genetic models (T vs. C: P = 0.453; T/T vs. C/C: P = 0.951; T/C vs. C/C: P = 0.534; T/T+T/C vs. C/C: P = 0.508; T/T vs. T/C+C/C: P = 0.246).

## Discussion

High myopia is a complex eye disease affected by both genetic and environmental factors, as well as gene-environment interactions [24], [25]. While the exact mechanism underlying this abnormal ocular development is still unclear, there is genomic and clinical evidence in various ethnic populations that genetics plays an important role in its development [7], [25]. In recent years, the rs3759223 and rs3759222 SNPs in the LUM gene have been widely tested for an association with high myopia, but the results remain controversial. Thus, this meta-analysis was conducted.

In the present study, the pooled results indicated that rs3759223 was associated with high myopia in a recessive model. Those with the TT genotype were at a 1.71-fold higher risk of high myopia than those with thee CC genotype and the TC genotype. With respect to rs3759222, there was no proof could verify the significant association between rs3759222 polymorphism and high myopia. Moreover, the sensitivity analysis revealed that the results were robust. Our meta-analysis suggests that LUM gene polymorphisms are associated with the development of high myopia.

The LUM gene is a member of SLRP gene family [11]. Proteoglycans are major components of the scleral extracellular matrix. These small proteoglycans play an important role in regulating collagen fibril assembly and interaction and are intensely related to the structure and function of the sclera [26]. In LUM-null zebrafish, the collagen fibrils were thinner, and the spatial distribution of the fibrils was less well-organized than in wild type zebrafish [27]. The two SNPs (rs3759222 and rs3759223) were in the promoter regions of the LUM gene. These two SNPs could result in a change in the putative regulatory elements, without any change in the codon, which influences the promoter activities of lumican and the level of expression of LUM mRNA [28]. This may cause significant defects in the scleral extracellular matrix, which could result in alterations in ocular shape and size.

Our subgroup analysis by study design showed that hospital-based studies yielded more significant association signals than population-based studies. Generally, in population-based studies, it is not clear whether the people in controls have other diseases that could exert a confounding effect on the true association. One potential explanation is that controls in the HB subgroup may merely represent a sample of people with eye diseases and that most of the patients have refractive error. Thus, the prevalence of myopia, even high myopia, may be higher than in the community population, and the results of the study design subgroup analysis should be interpreted cautiously.

Heterogeneity is a potential problem that may affect the interpretation of the results. In our meta-analysis, significant heterogeneity was detected in some comparisons. To eliminate heterogeneity, we carried out a subgroup analysis and used a random-effects model to pool the results whenever significant heterogeneity was present. Substantial heterogeneity was observed in the analysis of rs3759223 polymorphism, which was not surprising given the differences in the characteristics of the populations and genotyping methods. Our sensitivity analyses suggest that the study conducted by Zhang and colleagues [16] contributed to the heterogeneity. The small number of cases and participants in this study increased the possibility that chance accounted for the results.

While the current study was in progress, Feng et el [29] and Deng et el [30] as well as Liao et al [31] reported similar researches concern the relationship between rs3759223 polymorphisms and high myopia. Our meta-analysis is still superior over them in some aspects even though there are some similarities between our research and those. As to the included studies concern the relationship between rs3759223 polymorphism and high myopia, two of these included five studies and one included seven studies, which included fewer studies than our meta-analysis. Those three papers also have inconsistent results of the relationship between rs3759223 polymorphism and high myopia. Among these, Feng and Liao both held the view that this positive result exist in recessive model which is consistent with our study , however Deng concluded a negative outcome between rs3759223 polymorphism and high myopia. Besides there are no subgroup analysis in those research. In view of the conflicting evidence, there is necessity for us to conduct a meta-analysis. Furthermore our research analyzed two kinds of SNP polymorphisms (rs3759223 and rs3759222) within Lumican gene which is more comprehensive than those.

Some limitations of this meta-analysis should be addressed. Firstly, high myopia is a multi-factorial disease that results from complex interactions between various genetic and environmental factors. Our results were based on unadjusted estimates; a more precise analysis of the various groups should be conducted according to other factors, such as age and sex [32], [33]. Secondly, this meta-analysis was limited by the number of cases and controls, as well as small sample size, especially in the subgroup analysis. All of the included studies were carried out on Asians. Thus, the results may be applicable only to these ethnic populations. Thirdly, controls were not uniformly defined. This study is a meta-analysis of case-control studies. Only five were population-based. Thus, some selection bias may exist in the results, and they may not be representative of the general population. Fourthly, all included studies used a case-control design, which precludes further comments on the cause-effect relationship. Finally, the existing literature lacks information on potential gene-gene and gene-environment interactions [34]. Given that the roles of several environmental factors in the pathogenesis of myopia have been established, further research in this direction should be performed.

Despite of these limitations, this study also has some advantages. First, to minimize the bias within our research, we did not use the language limitation option when searching the literature databases, and all previous studies that met our criteria were included. In addition, the methodological issues regarding meta-analysis, such as heterogeneity, publication bias, and the stability of the results were all well-investigated. Moreover, our results were robust because the results of the sensitivity analysis were not materially altered and did not draw different conclusions.

In conclusion, this meta-analysis provides evidence that LUM polymorphism is associated with an increased risk of high myopia. Patients with the rs3759223 variants may have somewhat higher risks of developing high myopia as compared with controls. However, to reach a definitive conclusion, well-designed studies with larger sample sizes and more ethnic groups should be considered in order to further clarify the association. Moreover, gene-gene and gene-environment interaction studies should also be considered in future meta-studies.

## Supporting Information

Checklist S1 PRISMA checklist.
(DOC)
